# PRO-P: evaluating the effect of electronic patient-reported outcome measures monitoring compared with standard care in prostate cancer patients undergoing surgery—study protocol for a randomized controlled trial

**DOI:** 10.1186/s13063-024-08579-8

**Published:** 2024-11-12

**Authors:** Rouvier Al-Monajjed, Peter Albers, Johanna Droop, Dominik Fugmann, Joachim Noldus, Rein-Jüri Palisaar, Manuel Ritter, Jörg Ellinger, Philipp Krausewitz, Michael Truß, Boris Hadaschik, Viktor Grünwald, Andres-Jan Schrader, Philipp Papavassilis, Nicole Ernstmann, Barbara Schellenberger, Anna Moritz, Christoph Kowalski, Martin Hellmich, Pierce Heiden, Anna Hagemeier, Dirk Horenkamp-Sonntag, Markus Giessing, Luis Pauler, Sebastian Dieng, Maria Peters, Günter Feick, André Karger, PRO-P study group, Rouvier Al-Monajjed, Peter Albers, Johanna Droop, Dominik Fugmann, Joachim Noldus, Rein-Jüri Palisaar, Manuel Ritter, Jörg Ellinger, Philipp Krausewitz, Michael Truß, Boris Hadaschik, Viktor Grünwald, Andres-Jan Schrader, Philipp Papavassilis, Nicole Ernstmann, Barbara Schellenberger, Anna Moritz, Christoph Kowalski, Martin Hellmich, Pierce Heiden, Anna Hagemeier, Dirk Horenkamp-Sonntag, Markus Giessing, Luis Pauler, Sebastian Dieng, Maria Peters, Günter Feick, André Karger, Isabelle Bleiziffer, Isabelle Bußhoff, Franziska Winterhagen, Alix Tschirhart, Franziska Knappe, Caterina Shiminazzo, Julia Dung, Chantal Oberbeck, Sonja Seidemann, Sabine Würdig, Studienzentrale, Christopher Darr, Claudia Kesch, Tanja Brinkforth, Fereshteh Sadeghi Shakib, Maria Echterhoff, Litha Raubach, Marleen Greese-Turki, Julia Neumann, Julia Cornelia Frehse, Nils Jakob Michaelis, Carsten Schwarzer, Luis Linda Busse, Patricia Rausch, Matteo Silberg, Katja Fritz, Giulia Giersbach, Meike Mohr, Stefan Wiedelmann, Kerstin Voitz, Christiane Bothe, Fabian Queißert, Helga Flaswinkel, Kerstin Hermes-Moll

**Affiliations:** 1 Department of Urology, Medical Faculty and University Hospital Düsseldorf, Heinrich Heine University Düsseldorf, Germany and Center for Integrated Oncology Aachen, Bonn, Cologne, Düsseldorf (CIO-ABCD, Germany), Düsseldorf, Germany; 2 Clinical Institute for Psychosomatic Medicine and Psychotherapy, Medical Faculty and University Hospital Düsseldorf, Heinrich Heine University Düsseldorf, Germany and Center for Integrated Oncology Aachen, Bonn, Cologne, Düsseldorf (CIO-ABCD, Germany), Düsseldorf, Germany; 3 Department of Urology, Marien Hospital Herne, Ruhr-University Bochum, Bochum, Germany; 4 Department of Adult and Pediatric Urology, University Hospital Bonn, Bonn, Germany and Center for Integrated Oncology Aachen, Bonn, Cologne, Düsseldorf (CIO-ABCD, Germany), Bonn, Germany; 5 Department of Urology, Klinikum Dortmund, Dortmund, Germany; 6 Department of Urology, University Hospital Essen, Essen, Germany; 7 Department of Adult and Pediatric Urology, University Hospital Münster, Münster, Germany; 8 Institute of Medical Sociology, Health Services Research, and Rehabilitation Science (IMVR), Chair of Health Services Research, Faculty of Medicine and University Hospital Cologne, University of Cologne, Cologne, Germany; 9 Department of Certification - Health Services Research, German Cancer Society, Berlin, Germany; 10 Institute of Medical Statistics and Computational Biology (IMSB), Medical Faculty and University Hospital Cologne, University of Cologne, Cologne, Germany; 11 Techniker Krankenkasse, Hamburg, Germany; 12 Department of Urology, Kliniken Maria Hilf, Mönchengladbach, Germany; 13 OnkoZert GmbH, Neu-Ulm, Germany; 14 Division of Personalized Early Detection of Prostate Cancer, German Cancer Research Center (DKFZ), Heidelberg, Germany; 15 AOK Rheinland/Hamburg, Düsseldorf, Germany; 16 Federal Prostate Cancer Self-Help, BPS, Bonn, Germany; 17 Department for Medical Oncology, University Hospital Essen, Essen, Germany

**Keywords:** Prostate cancer, PROM, Continence, EPIC-26, Patient-reported outcomes, Quality of life, Health apps, Radical prostatectomy

## Abstract

**Background:**

With over 65,000 new cases per year in Germany, prostate cancer (PC) is the most common cancer in men in Germany. Localized PC is often treated by radical prostatectomy and has a very good prognosis. Postoperative quality of life (QoL) is significantly influenced by the side effects of surgery. One possible approach to improve QoL is postoperative symptom monitoring using ePROMs (electronic patient-reported outcome measures) to accurately identify any need for support.

**Methods:**

The PRO-P (“Influence of ePROMS in surgical therapy of PC on the postoperative course”) study is a randomized controlled trial employing 1:1 randomization at 6 weeks postoperatively, involving 260 patients with incontinence (≥ 1 pad/day) at six participating centers. Recruitment is planned for 1 year with subsequent 1-year follow-up. PRO-monitoring using domains of EPIC-26, psychological burden, and QoL are assessed 6, 12, 18, 24, 36, and 52 weeks postoperatively. Exceeding predefined PRO-score cutoffs triggers an alert at the center, prompting patient contact, medical consultation, and potential interventions. The primary endpoint is urinary continence. Secondary endpoints refer to EPIC-26 domains, psychological distress, and QoL. Aspects of feasibility, effect, and implementation of the intervention will be investigated within the framework of a qualitative process evaluation.

**Discussion:**

PRO-P investigates the effect on postoperative symptom monitoring of a structured follow-up using ePROMs in the first year after prostatectomy. It is one of the first studies in cancer surgery investigating PRO-monitoring and its putative applicability to routine care. Patient experiences with intensified monitoring of postoperative symptoms and reflective counseling will be examined in order to improve primarily urinary continence, and secondly other burdens of physical and psychological symptoms, quality-of-life, and patient competence. The potential applicability of the intervention in clinical practice is facilitated by IT adaption to the certification standards of the German Cancer Society and the integration of the ePROMs survey via a joint patient portal. Positive outcomes could readily translate this complex intervention into routine clinical care. PRO-P might improve urinary incontinence and QoL in patients with radical prostatectomy through the structured use of ePROMs.

**Trial registration:**

ClinicalTrials.gov NCT05644821. Registered on 09 December 2022.

**Supplementary Information:**

The online version contains supplementary material available at 10.1186/s13063-024-08579-8.

## Administrative information

Note: the numbers in curly brackets in this protocol refer to SPIRIT checklist item numbers. The order of the items has been modified to group similar items (see http://www.equator-network.org/reporting-guidelines/spirit-2013-statement-defining-standard-protocol-items-for-clinical-trials/).

**Table:** 

Title {1}	PRO-P: evaluating the effect of electronic patient-reported outcome measures monitoring compared with standard care in prostate cancer patients undergoing surgery—study protocol for a randomized controlled trial
Trial registration {2a and 2b}.	ClinicalTrials.gov. Registration number: NCT05644821. Registered on 09 December 2022.
Protocol version {3}	Version 2.3, date 10/05/2023
Funding {4}	The Federal Joint Committee (Gemeinsamer Bundesausschuss, G-BA), Germany, grant number 01VSF21034)
Author details {5a}	Rouvier Al-Monajjed^1^, Peter Albers^1,14^, Johanna Droop^1^, Dominik Fugmann^2*^, Joachim Noldus^3^, Rein-Jüri Palisaar^3^, Manuel Ritter^4^, Jörg Ellinger^4^, Philipp Krausewitz^4^, Michael Truß^5^, Boris Hadaschik^6^, Viktor Grünwald^6,17^, Andres-Jan Schrader^7^, Philipp Papavassilis^7^, Nicole Ernstmann^8^, Barbara Schellenberger^8^, Anna Moritz^8^, Christoph Kowalski^9^, Martin Hellmich^10^, Pierce Heiden^10^, Anna Hagemeier^10^, Dirk Horenkamp-Sonntag^11^, Markus Giessing^12^, Luis Pauler^13^, Sebastian Dieng^13^, Maria Peters^15^, Günter Feick^16^, André Karger^2^ **PRO-P study group:** (see appendix for all members) ^1^Department of Urology, Medical Faculty and University Hospital Düsseldorf, Heinrich Heine University Düsseldorf, Germany and Center for Integrated Oncology Aachen, Bonn, Cologne, Düsseldorf (CIO-ABCD, Germany) ^2^Clinical Institute for Psychosomatic Medicine and Psychotherapy, Medical Faculty and University Hospital Düsseldorf, Heinrich Heine University Düsseldorf, Germany and Center for Integrated Oncology Aachen, Bonn, Cologne, Düsseldorf (CIO-ABCD, Germany) ^**3**^Department of Urology, Marien Hospital Herne, Ruhr-University Bochum, Germany ^4^Department of Adult and Pediatric Urology, University Hospital Bonn, Bonn, Germany and Center for Integrated Oncology Aachen, Bonn, Cologne, Düsseldorf (CIO-ABCD, Germany) ^5^ Department of Urology, Klinikum Dortmund, Dortmund, Germany ^6^Department of Urology, University Hospital Essen, Essen, Germany ^7^Department of Adult and Pediatric Urology, University Hospital Münster, Münster, Germany ^8^Chair of Health Services Research, Institute of Medical Sociology, Health Services Research, and Rehabilitation Science, Faculty of Medicine and University Hospital Cologne, University of Cologne, Cologne, Germany ^9^Department of Certification—Health Services Research, German Cancer Society, Berlin, Germany ^10^Institute of Medical Statistics and Computational Biology (IMSB), Medical Faculty and University Hospital Cologne, University of Cologne, Germany ^11^Techniker Krankenkasse, Hamburg, Germany ^12^Department of Urology, Kliniken Maria Hilf, Mönchengladbach, Germany ^13^OnkoZert GmbH, Neu-Ulm, Germany ^14^Division of Personalized Early Detection of Prostate Cancer, German Cancer Research Center (DKFZ), Heidelberg, Germany ^*15*^AOK, Rheinland/Hamburg, Germany ^16^ Federal Prostate Cancer Self-help, BPS, Bonn, Germany ^17^ Department for Medical Oncology, University Hospital Essen, Essen, Germany *Corresponding author Dr. med. Dominik Fugmann Adress: Universitätsklinikum Düsseldorf, Moorenstr. 5, 40225 Düsseldorf, Germany Email: Dominik.fugmann@med.uni-duesseldorf.de, Tel. + 49 211 81 17,354
Name and contact information for the trial sponsor {5b}	Heinrich Heine University, Düsseldorf, Germany
Role of sponsor {5c}	The study sponsor and funder do not have any authority over the study design; collection, management, analysis, and interpretation of data; writing of the report; and the decision to submit the report for publication.

## Introduction

### Background and rationale {6a}

With an annual incidence of over 65,000 new cases, prostate cancer stands as the most prevalent malignancy affecting men in Germany. With almost 15,000 deaths annually, it is the second most common tumor-associated cause of death in men. The 5- and 10-year survival rates for localized prostate cancer are generally good, at 89% and 88%, respectively, but depend on the initial tumor stage [1]. Curative treatment options for localized prostate cancer include surveillance, radical prostatectomy, and radiotherapy [2–4].

In patients with a limited life expectancy, symptom-oriented therapy (watchful waiting) remains a viable option [2]. Some subgroups, depending on the severity of the disease, the therapy chosen, and the age of the patient, suffer from long-term impairment of quality of life and health [5, 6]. Postoperatively, urinary incontinence and therapy-induced erectile dysfunction affect a substantial proportion of patients [7, 8]. Furthermore, increased psychological distress as well as increased anxiety or depression are also reported [9–11]. In the first 6 months after diagnosis of low-risk PC, the risk of suicide in PC patients is increased fivefold compared with the general population [12]. Several studies have shown that early intervention to improve continence is successful [13].

The German prostate cancer guideline recommends multidisciplinary rehabilitation using multimodal therapy concepts [2, 14, 15]. However, the need for supportive care is inadequately addressed by standardized care pathways in the treatment of PC [16]. Target group-specific services are largely lacking [17], and subjective information provided by the patient is also rarely systematically recorded and linked to appropriate interventions during routine follow-up and aftercare. In the medium- to longer-term follow-up, there is a lack of cross-provider shared-care and survivorship-care plans [18, 19]. Patient-reported outcomes (PROs) refer to “any report of a patient’s health status that comes directly from the patient (i.e. without interpretation by a physician or other person)” [20]. PROs can therefore describe symptoms that affect quality of life, functioning, or functional limitations, such as pain, loss of appetite, or erectile function. PROs are measured with patient-reported outcome measures [21], usually using standardized and broadly validated questionnaires. In oncology, PROMs are now used for quality assurance of care as well as treatment planning and monitoring of patients [22]. The effective use of PROMs to monitor patients in active therapy, as well as in follow-up, has now been demonstrated in a number of tumor types, especially for treatment with chemotherapy [23–29]. Electronic collection and use of PROMs are forward-looking [30, 31]. In this way, routine data can be processed in real time and integrated into the electronic health record. In the treatment of PC, PROMs provide important information on physical functioning, psychological well-being, and quality of life [32]. To date, PROMs have been used to compare the quality of care of different German Cancer Society-certified prostate cancer centers as part of the Prostate Cancer Outcome (PCO) study [33–36].

### Objectives {7}

In addition to quality-of-care monitoring, PRO-P utilizes ePROMs for individualized patient care. The PRO-P study is an innovative model for a care pathway in terms of intensity (frequency of symptom retrieval), real-time monitoring with the eHealth App, and optimized cross-provider interaction. In contrast to the existing standard of care, real-time detection, timely consultation, and treatment of postoperative symptoms could lead to reduced physical and psychological symptom burden as well as improved quality of life. Another consequence could be improved physician–patient communication. Intensified and standardized follow-up could strengthen patients’ ability to act independently to promote and maintain their health. This health care pathway will be transferable to the outpatient setting in the future.

PRO-P investigates the influence of a structured follow-up using ePROMs in the first year after prostatectomy on the postoperative course. The study explores whether early detection of symptoms through this intervention, followed by subsequent measures, results in improved outcomes related to incontinence, symptom burden, quality of life, and patient competence. Notably, the study evaluates the overall impact of a complex intervention integrating intensified ePROM monitoring with additional measures at the treatment center, rather than testing specific interventions.

### Trial design {8}

PRO-P is a multicenter, prospective, and two-arm 1:1 randomized controlled study.

**Fig. 1:**
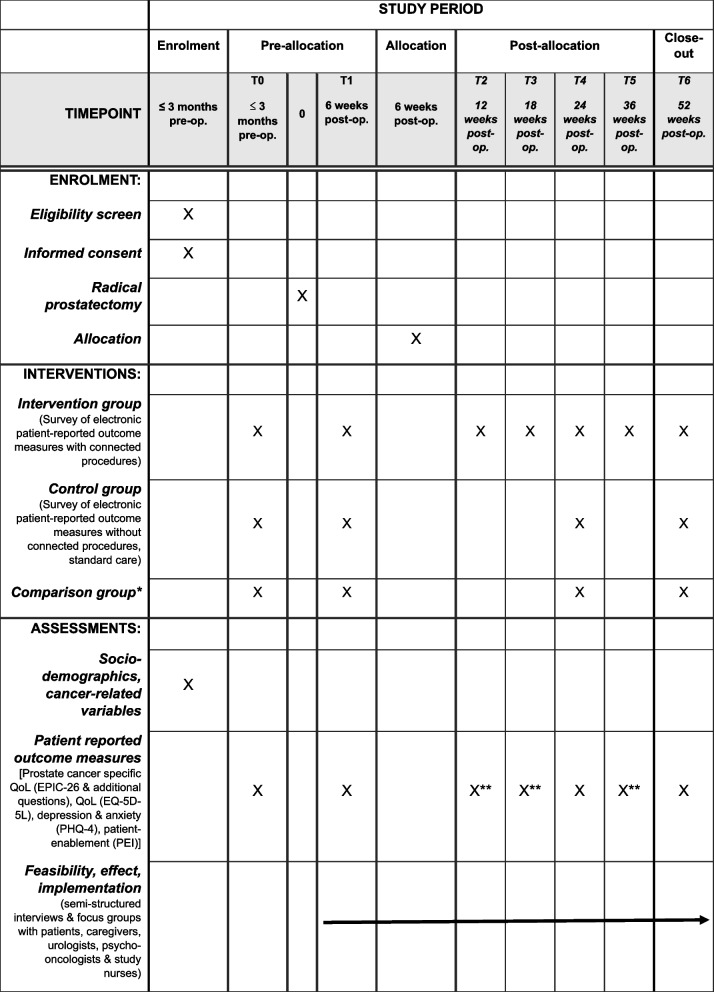
SPIRIT figure. Pre-op. = pre-operative; post-op. = post-operative; QoL = quality of life. *Comparison group: patients who are 6 weeks postoperatively urinary continent; **Survey of patients in the intervention group only

Randomization, stratified by study center (6 centers) and age (< / ≥ 70), is performed 6 weeks after surgery and directly linked to the first post-surgery survey. Patients with symptoms of urinary incontinence (≥ 1 pad/day, 6 weeks after surgery) will be randomized. Patients with no symptoms of urinary incontinence (0 pads/day 6 weeks after surgery) will be assigned to the comparison group (see Fig. 1).

Patients in the intervention group will be offered medical support, if defined thresholds of symptom burden are exceeded (e.g. at least one minimally important difference (MID) for EPIC domains). ePROMs in the control group will not trigger proactive study intervention and patients will receive standard-of-care management according to local guidelines. ePROM measures in the control group will serve as a reference for the trial population.

## Methods: participants, interventions, and outcomes

### Study setting {9}

The study is being conducted at six German certified cancer centers (five university hospitals and one academic teaching hospital):

- Department of Urology, University Hospital Düsseldorf, Düsseldorf, Germany
- Department of Urology, Marien Hospital Herne, Ruhr-University Bochum, Herne, Germany
- Department of Adult and Pediatric Urology, University Hospital Bonn, Bonn, Germany
- Department of Urology, Klinikum Dortmund, Dortmund, Germany
- Department of Urology, University Hospital Essen, Essen, Germany
- Department of Adult and Pediatric Urology, University Hospital Münster, Münster, Germany

### Eligibility criteria {10}

Eligible participants must be aged 18 years or older, male, and diagnosed with prostate cancer (TNM T1-4 NX N0-1 M0-1c, no relapse). They must be scheduled for a primary radical prostatectomy (excluding salvage operations). Additionally, participants must possess legal capacity, possess sufficient proficiency in the German language, and have access to a mobile digital device or desktop computer. They should demonstrate the capability to receive emails or push notifications online and possess the ability to independently complete electronic questionnaires, with guidance or assistance if required.

Participants meeting any of the following criteria will be excluded from the study: those in a palliative treatment situation with a life expectancy of less than 1 year, individuals with preoperative urinary incontinence, individuals with existing urinary diversion, and/or those scheduled for a planned cystectomy.

### Who will take informed consent? {26a}

Upon verification of eligibility criteria and obtaining written informed consent by a study physician, patients will be enrolled in the study, either during the prostate cancer outpatient appointment prior to treatment or upon hospital admission for radical prostatectomy.

### Additional consent provisions for collection and use of participant data and biological specimens {26b}

N/a. No additional consent provision is required. There will be no biological specimens collected.

## Interventions

### Explanation for the choice of comparators {6b}

In the control group, ePROMs will be collected 6, 24, and 52 weeks after radical prostatectomy, but without further measures. Therefore, they will receive treatment according to the current clinical routine [2].

### Intervention description {11a}

**Fig. 2:**
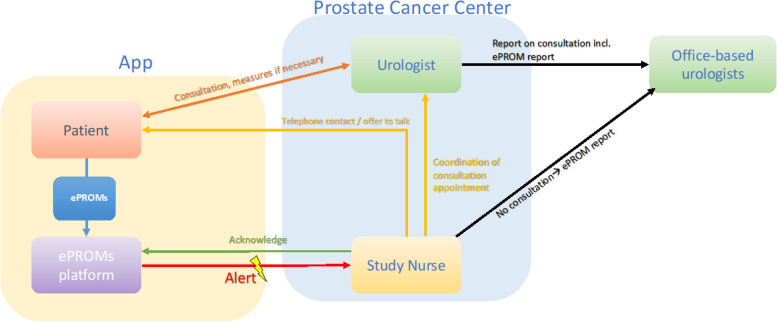
Schematic representation of the intervention

An alert triggered by the EPIC-26 domains and the PHQ-4 will result in contact from the personnel at the prostate cancer center with the intervention group (see Fig. 2). For EPIC-26, an alert is triggered if the preoperative score drops by at least one minimally important difference (MID) in at least one domain [37]. This is based on the age-adjusted, anchor-based MID. Thresholds for the 5 EPIC-26 domains are set for urinary incontinence (≥ 9 points), irritative/obstructive symptoms (≥ 7 points), gastrointestinal symptoms (≥ 5 points), sexual interest (≥ 10 points), and hormonal function/vitality (≥ 4 points). For the subscales of PHQ-4, cutoff points are defined for depression (≥ 3 points) and generalized anxiety (≥ 3 points) whose attainment (independent of the preoperative score) triggers an alert at each interview time point.

In the intervention group, ePROMs (EPIC-26, PHQ-4) are obtained after 6, 12, 18, 24, 36, and 52 weeks. If cut-off values are exceeded, an alert is triggered at the prostate cancer treatment center (PCC). In order to ensure that ePROMs are filled in within a defined time-frame, ePROM statuses will be closely monitored by the study nurses. In addition to electronic reminders, patients will be reminded by study nurses via phone calls in a structured manner.

- ➔ An alert is dealt with by a study nurse who makes a standardized telephone contact with the patient.
- ➔ Asking about symptoms and offering to coordinate consultation with a trained urologist at the PCC. Content of the consultation: a medical history, clinical examination, and detailed consultation, as well as, if necessary, the guideline-compliant initiation of further diagnostics and therapy, including follow-up contacts necessary in this context.
- ➔ Before consultation, urologists receive a written summary and visualization of the ePROM test results in the clinic information system.

In addition, postal communication of a report on the results of a urological consultation at the PCC or, if the patient has either not indicated a need for consultation in the telephone contact with the study nurse or has not been reached by the study nurse, of the ePROM results to the outpatient urologist.

### Criteria for discontinuing or modifying allocated interventions {11b}

- No primary radical prostatectomy performed
- Change of center to a prostate cancer center not participating in the study
- Patient request
- Patient is no longer able to participate
- Increased psychological stress induced by the ePROM survey (clarification of causes in the consultation), which cannot be alleviated despite consultations and further measures

### Strategies to improve adherence to interventions {11c}

Patients receive email reminders and, if using the app, push notifications when a new questionnaire has been assigned for the ePROM survey. If questionnaires are still not completed, up to three telephone contact attempts are made as a reminder.

### Relevant concomitant care permitted or prohibited during the trial {11d}

There are no relevant concomitant care and interventions that are permitted or prohibited during the trial.

### Provisions for post-trial care {30}

No provision is provided.

### Outcomes {12}

#### Summative evaluation

##### Primary outcome

To calculate the primary endpoint, the difference is calculated between the scores on the urinary incontinence scale of the EPIC-26 [38] at 52 and 6 weeks postoperatively. Changes regarding urinary incontinence (minimally important difference (MID) ≥ 9) at 52 weeks postoperatively compared with 6 weeks postoperatively are considered. The primary endpoint will be examined in an intention-to-treat analysis. The comparison regarding superiority of the primary endpoint will be performed by using a mixed linear model for repeated measures. The variables included in the model comprise intervention/control group, time, group*time, center, tumor stage, and age group.

##### Secondary outcomes

Secondary outcomes comprise all other domains of the EPIC-26 [38] with the according MID changes (sexual function, MID ≥ 10; irritative/obstructive symptoms, MID ≥ 7; gastrointestinal symptoms, MID ≥ 5; vitality/hormonal function, MID ≥ 4) [37], changes in health-related quality of life according to the EQ-5D-5L [39], changes in depression and generalized anxiety according to the PHQ-4 [40] as well as in changes to patient enablement according to the PEI [41].

### Process evaluation

According to the Medical Research Council Guidance on the development and evaluation of complex interventions [42], a feasibility analysis, an impact analysis, and an investigation of change processes, as well as an analysis of the implementation quality will be carried out in the corresponding project phases as part of a process evaluation. A qualitative process evaluation in three modules will be performed. The three modules are as follows: feasibility, impact, and implementation. The evaluation will be done by semi-structured interviews with patients (and relatives), urologists, psycho-oncologists, and study nurses. Patients from the intervention group and the control group are interviewed at two time points (approximately 12 weeks and approximately 52 weeks after surgery). The data are qualitatively analyzed using content analytic methods. Criteria for Module 1 are based on the feasibility criteria [43], for assessing the acceptance and feasibility of the intervention and study procedures. Analysis of implementation factors in Module 3 follows the Consolidated Framework for Advancing Implementation Science (CFIR) [44].

Thus, the results of the effectiveness study can be explained and the effective components of the intervention can be described. In the context of later implementation and dissemination, the benefit of the intervention—adapted on the basis of the program theory—can be further increased.

### Participant timeline {13}

**Fig. 3:**
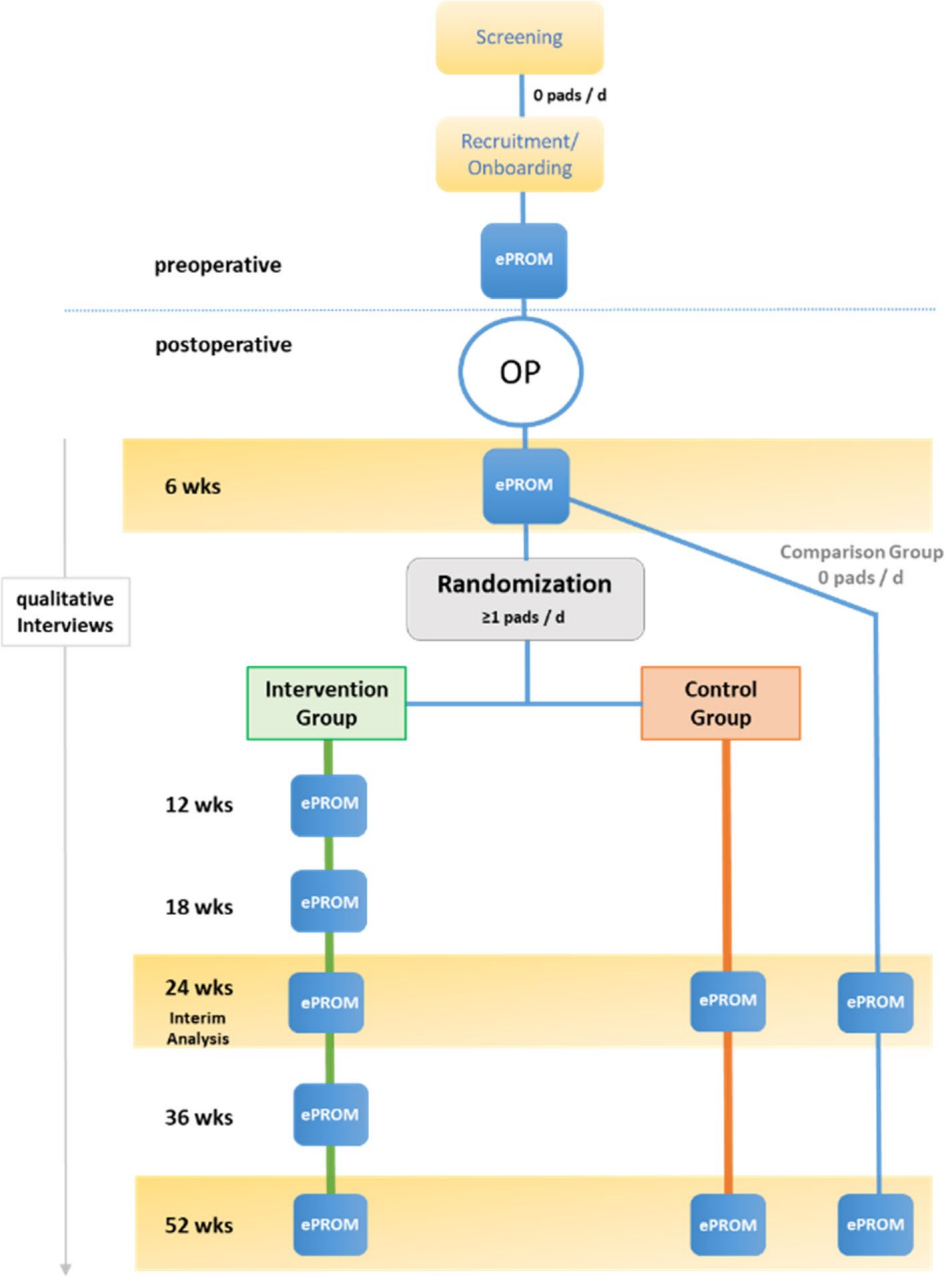
Study design and participant timeline

EPROMs will be issued in a standardized fashion once before and six times (intervention group) or three times (control group) after radical prostatectomy in patients with localized PC (see Fig. 3).

### Sample size {14}

**Fig. 4:**
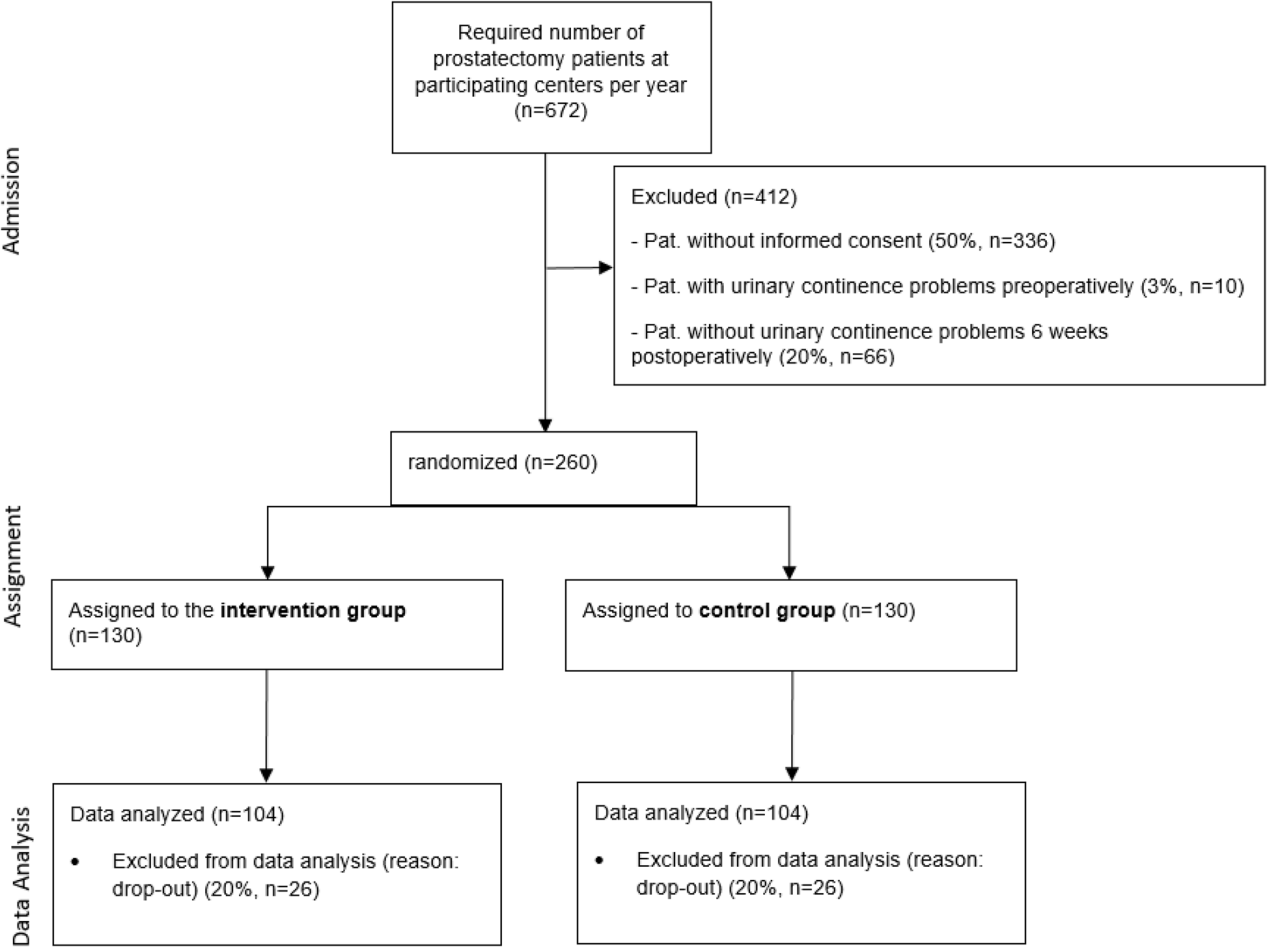
Consort data flow

Based on the information that preoperative urinary continence issues are observed in 3% [35] and a continence rate of 20% (0 pad/day) is anticipated 6 weeks postoperatively [7], the required sample size is calculated as follows: A total of 672 patients who undergo prostatectomy at participating centers will be considered for inclusion in the study. Of these, 412 are expected to be excluded for the following reasons: 336 patients (50%) will not provide informed consent, 10 patients (3%) will have preoperative urinary incontinence, and 66 patients (20%) will show no urinary incontinence 6 weeks postoperatively. The remaining 260 patients will be randomized, with 130 assigned to the intervention group and 130 to the control group. Data analysis will be conducted for 104 patients in each group, with 26 patients (20%) anticipated to be excluded from analysis due to dropout (see Fig. 4).

To detect a mean effect size of 0.4 (Cohen’s *d*) for the primary endpoint, 99 patients per group are required, assuming 80% power and a 5% two-sided error.

### Recruitment {15}

All prostate cancer patients at the participating study centers will be screened for eligibility by study physicians, either during the outpatient prostate cancer consultation or upon hospital admission for radical prostatectomy. If eligible, patients will be offered participation in the study and informed consent will be obtained. The recruitment period will last for 1 year. It is expected that, on average, 2.3 patients per center will be randomized in the first month. In months 2 through 4, this number is anticipated to increase to 2.9 patients per center, followed by 3.1 patients per center in months 5 through 7. For months 8 through 10, the expectation is 4.2 patients per center, and finally, 4.8 patients per center during months 11 and 12.

The recruitment target calculated above is realistic, as approximately 1200 men undergo prostatectomy annually in the six participating prostate cancer treatment centers, of which we expect to reach up to 770 patients.

## Assignment of interventions: allocation

### Sequence generation {16a}

Randomization will be performed by allocating patients in a 1:1 ratio based on permuted blocks, stratified by center and age group (< / ≥ 70).

### Concealment mechanism {16b}

Randomization will be performed centrally using ALEA’s 24/7 internet service.

### Implementation {16c}

Study nurses at the respective study center assign the participants to the intervention or control arm depending on the randomization result in the survey software.

## Assignment of interventions: blinding

### Who will be blinded {17a}

Due to the nature of the intervention, blinding is not possible for either participants or care providers. Outcome assessment is undertaken by the patients. Data analysts will not be blinded.

### Procedure for unblinding if needed {17b}

N/a.

The design is open label so unblinding will not occur.

## Data collection and management

### Plans for assessment and collection of outcomes {18a}

The patients complete their questionnaires either via a web-based platform or using an ePROM app.

Instruments: The Expanded Prostate Cancer Index Short Form (EPIC-26) is a questionnaire designed to evaluate the symptoms and functions of individuals with prostate cancer [37, 38]. Comprising 26 individual items, the instrument assesses five distinct domains: urinary incontinence, irritative/obstructive symptoms, gastrointestinal symptoms, sexual function, and hormonal function/vitality. Each scale is scored out of 100 points, with higher scores indicating superior function. Preoperatively, additional questions on sexual interest [45], use of sexual aids [46], nationality, insurance status, and education are integrated [34]. In addition, questions are asked about occupational status, marital status, presence of children (especially minor children) in the household, previous psychological or psychiatric treatment, and current psychotropic drug therapy.

Postoperatively, additional questions on sex from the pre-therapeutic supplementary questions, tumor status (PSA value, recurrence/lymph node recurrence, distant metastases, second tumor), subjective treatment success, use of rehabilitation measures, and one on urinary diversion by means of a urinary bladder catheter or insertion of a ureteral splint are posed at each survey time point. After 52 weeks, all participating patients will be asked about the use of assistance after prostate cancer surgery (patient guidebooks or guidelines, physiotherapy, psycho-oncological counseling, psychotherapy, self-help group, social worker/social service, pastoral care).

The Patient Health Questionnaire-4 (PHQ-4) measures symptoms of depression and generalized anxiety on two subscales with two items each [40]. Scores from 0 to 3 can be obtained on each question, so that a cumulative score of 12 can be obtained, with a higher score indicating more pronounced symptomatology.

The European Quality of Life Five-Dimension questionnaire (EQ-5D-5L) measures health-related quality of life on five scales: mobility, ability to care for oneself, activities of daily living, pain/physical discomfort, and anxiety/depression [39]. Each scale is represented by a question that provides five response options for graduating symptomatology. In addition, a question on self-assessment of health status is asked on a visual analog scale from 0 to 100, where 100 is the best imaginable health.

The Patient Enablement Instrument (PEI) measures patient empowerment through six questions [41]; a modified question sequence and Likert scale are used. The questions map the extent to which patients feel empowered to understand their health problems or illness, manage their health problems/illness, maintain their health, manage their lives, trust their health, and help themselves. Overall, the sum score ranges from 6 to 30 points, with a higher score corresponding to greater patient empowerment (see Table 1).

**Table 1:** Instruments

Name	Domains
Expanded Prostate Cancer Index Short Form (EPIC-26)	- Urinary incontinence - Irritative/obstructive symptoms - Gastrointestinal symptoms - Sexual function - Hormonal function/vitality
Patient Health Questionnaire-4 (PHQ-4)	- Depression and generalized anxiety
European Quality of Life Five-Dimension questionnaire (EQ-5D-5L)	- Health-related quality of life on five scales: mobility, ability to care for oneself, activities of daily living, pain/physical discomfort, and anxiety/depression
Patient Enablement Instrument (PEI)	- Patient enablement
Additional questions in analogy to the PCO study	- Interest in sex [42] - Use of sexual aids [43] - Nationality - Insurance status - Education
Additional questions	- Occupational status - Marital status, presence of children (especially minor children) in the household - Previous psychological or psychiatric treatment - Current psychotropic drug therapy

### Plans to promote participant retention and complete follow-up {18b}

Study participants are reminded to complete questionnaires by email and push notifications. Subsequently, patients are contacted by telephone and reminded to complete questionnaires.

Deviations from the study protocol are noted in the study database.

Study participants whose data sets are no longer usable in the context of the study due to deviations from the study protocol are assigned to a third group, the comparison group, and are also surveyed after 1 year.

### Data management {19}

Disease- and treatment-related data will align with the “OncoBox Prostate” data structure in all German Cancer Society-certified prostate cancer centers.

Primary data collection: All primary cases are created by the participating clinics for the statutory cancer registration in the tumor documentation system (TuDok). The clinics then check whether the patient fulfills the inclusion criteria during a face-to-face contact with a study physician. If not, this is indicated in TuDok. If the inclusion criteria are met, the patient is informed about the study and asked for consent; the result is noted in the TuDok. A prerequisite for inclusion is that the patient has a digital device. If consent is given, the patient is included in the study and created in the ePROM application. The data is stored together with the patient's e-mail address in encrypted form in the TÜV-certified Telekom Health Care Cloud. The declaration of consent with name and address remains at the center. Study participants fill out questionnaires in the ePROM application. As soon as questionnaires are assigned, notifications are sent to the study participants via the ePROM application (push messages via an app and e-mail reminders). Questionnaires are transferred pseudonymized by means of a study ID from the survey software via an interface to the web-based study documentation Pro-P-Doc. As all clinical information (diagnosis and therapy) is already documented in the tumor documentation system at this point, this data can be transferred to the study documentation via the OncoBox Prostata (a current list of fields can be found in the OncoBox specification: http://www.xml-oncobox.de/de/Zentren/ProstataZentren) and merged with the pre- and post-therapeutic survey data. The study center also documents relevant study information such as (a) whether the patient continues to meet the inclusion criteria, (b) whether the patient was randomized to the intervention or control group, (c) whether and if so, which form of intervention was offered to the patient, and (d) whether the patient took advantage of it. The survey data can also be transferred from Pro-P-Doc to the hospital information system together with selected master and clinical data in the form of a pseudonymized (study ID) PDF; this is done manually by the study nurses at the center, who keep the pseudonymization keys so that a clear assignment to the electronic patient file is possible.

The Pro-P-Doc web application is provided on a server. The complete data set is retrieved from Pro-P-Doc at regular intervals. When patients are created in Pro-P-Doc, a consecutive ID (Pro-P-Doc ID) is assigned. In this step, the data is only pseudonymized using the PRO-P-Doc ID (removal of the study and OncoBox ID, deletion of personal data, age at first diagnosis instead of date of birth, Pro-P-Doc ID) and transferred to OncoBox Research Pro-P.

For qualitative research, contact forms containing patients’ real names, contact details, and study IDs are stored in a trust center on protected IMVR servers so that patients can be contacted directly for interviews. Interviews are pseudonymized by a trust center (study ID) and transcribed by external service providers. The audio recordings are then destroyed. All text passages that could be used to identify third parties are removed from the transcripts, so that the transcripts are pseudonymized in relation to the patients and anonymized in relation to third parties. In order to merge the interview data with the survey data (PDF from Pro-P Doc), the survey data can be requested from the study nurse of the center concerned, stating the study ID. The respective survey data set (PDF from Pro-P Doc) is then transmitted to the IMVR pseudonymized (study ID).

### Confidentiality {27}

Patient data is processed pseudonymized; see the “Data management” section.

### Plans for collection, laboratory evaluation, and storage of biological specimens for genetic or molecular analysis in this trial/future use {33}

N/a.

No biological specimens will be collected.

## Statistical methods

### Statistical methods for primary and secondary outcomes {20a}

#### Summative evaluation

##### Primary outcome

To calculate the primary endpoint, the difference is calculated between the scores on the urinary incontinence scale of the EPIC-26 at 52 and 6 weeks postoperatively. Changes regarding urinary incontinence (minimally important difference (MID) ≥ 9) at 52 weeks postoperatively compared with 6 weeks postoperatively are considered. The primary endpoint will be examined in an intention-to-treat analysis. The comparison regarding the superiority of the primary endpoint will be performed by using a mixed linear model for repeated measures. The variables included in the model comprise intervention/control group, time, group*time, center, tumor stage, and age group.

##### Secondary outcomes

Secondary outcomes comprise all other domains of the EPIC-26 with the according MID changes (sexual function, MID ≥ 10; irritative/obstructive symptoms, MID ≥ 7; gastrointestinal symptoms, MID ≥ 5; vitality/hormonal function, MID ≥ 4), changes in health-related quality of life according to the EQ-5D-5L, changes in depression and generalized anxiety according to the PHQ-4 as well as in changes to patient enablement according to the PEI.

### Interim analyzes {21b}

The study is scheduled to run for 36 months. Flanked by a 12-month pre-study and an 8-month follow-up, data collection will take place over a period of 28 months. This ensures that the required calculated number of cases can be safely achieved. A first interim analysis will be performed in a group sequential design with Haybittle-Peto barriers with interim analysis after 24 weeks, in order to make adaptations concerning the duration, the number of cases, and the design elements. Specifically, the treatment discontinuation (insufficient adherence) and the adequacy of the data collection forms will be evaluated in order to possibly adapt the study process.

### Methods for additional analyses (e.g., subgroup analyses) {20b}

#### Process evaluation

According to the Medical Research Council Guidance on the development and evaluation of complex interventions [42], a feasibility analysis, an impact analysis, and an investigation of change processes, as well as an analysis of the implementation quality will be carried out in the corresponding project phases as part of a process evaluation. A qualitative process evaluation in three modules will be performed. The three modules are: feasibility, impact, and implementation. The evaluation will be done by semi-structured interviews with patients (and relatives), urologists, psycho-oncologists, and study nurses. Patients from the intervention group and the control group are interviewed at two time points (approximately 12 weeks and approximately 52 weeks after surgery). The data are qualitatively analyzed using content analytic methods. Criteria for Module 1 are based on the feasibility criteria [43], for assessing the acceptance and feasibility of the intervention and study procedures. Analysis of implementation factors in Module 3 follows the Consolidated Framework for Advancing Implementation Science (CFIR) [44].

Thus, the results of the effectiveness study can be explained and the effective components of the intervention can be described. In the context of later implementation and dissemination, the benefit of the intervention—adapted on the basis of the program theory—can be further increased.

### Methods in analysis to handle protocol non-adherence and any statistical methods to handle missing data {20c}

The influence of missing values will be analyzed in sensitivity analyses (multiple imputation). To analyze the secondary outcome variables, mixed linear models for repeated measures will be used, analogous to the analysis of the primary endpoint. These analyses will be performed without controlling for multiple experimental errors.

### Plans to give access to the full protocol, participant-level data and statistical code {31c}

The full study protocol is available from the corresponding author. The participants in this study do not provide explicit consent for public data sharing. To protect their privacy, the data will not be available for public access. The data are available upon reasonable request, provided that the request is for research purposes, the confidentiality of the data is maintained, and appropriate ethical approval is demonstrated. Statistical code will be available from the IMSB, Cologne, Germany, upon reasonable request.

### Oversight and monitoring

#### Composition of the coordinating center and trial steering committee {5d}

The Steering Board is composed of the project managers of the sub-projects. Each partner is represented by at least one member of the Steering Board. The Steering Board advises and decides on matters of general or fundamental importance within the consortium. The Steering Board is advised by a patient representative from the Federal Association of Prostate Cancer Self-Help (Bundesverband Prostatakrebsselbsthilfe e.V.). The role of the patient representative is to bring in the patient perspective, ensure that the needs and concerns of patients are considered, and advocate for patient-centered approaches throughout the study. The tasks of the Steering Board members include in particular:

- Supervision of the implementation of the plans and objectives in the project,
- Monitoring the progress and development of the partners. This includes, in particular, compliance with the milestones specified in the joint application to the funding organization,
- Reviewing and commenting on planned publication projects, as well as counseling and arbitration in the event of disagreements, e.g., with regard to the naming and order of authors,
- Arbitration in the event of disagreements regarding the use of data from the project, and
- Decisions on the inclusion or exclusion of partners.

The project group is made up of the local study coordinators of the partners, along with a patient representative from the Federal Association of Prostate Cancer Self-Help (Bundesverband Prostatakrebsselbsthilfe e.V.). The project group meets once a month to discuss the progress and problems of the project.

The study nurse group is made up of the study nurses from all recruiting centers and the study coordinators from the consortium management and meets weekly to discuss practical problems in the study process.

### Composition of the data monitoring committee, its role and reporting structure {21a}

A DMC is not planned as part of the study, as only standard care is being compared to enhanced follow-up care—serious adverse events (SAEs) are therefore not expected. Regular monitoring of the data and reporting to all partners is carried out by OnkoZert (Neu-Ulm, Germany) after the start of recruitment. The content of the reporting will be discussed at the project group meetings and steering board meetings. Independent patient representatives are also part of the project group and the steering board in an advisory capacity.

### Adverse event reporting and harms {22}

In this study, we have developed a comprehensive plan for collecting, assessing, reporting, and managing both solicited and spontaneously reported adverse events (AEs) and any unintended effects related to the trial interventions or conduct. While the primary comparison involves enhanced follow-up care versus standard post-prostatectomy care, potential AEs such as increased psychological strain due to repeated questioning or the focus on the illness have been considered. To mitigate this, any participant reporting psychological distress will be offered a medical consultation. Additionally, technical issues during the survey process may also lead to participant frustration or stress, which has been identified as another potential AE. In such cases, participants are encouraged to contact the study nurses or study coordination team to report these issues and receive prompt assistance. All participants are provided with contact information for the study leadership, coordination team, and Study Nurses at their center, allowing them to report any AEs or concerns as they arise. The study nurses are in continuous communication with the study coordination team, which is available at all times for immediate support. To ensure ongoing monitoring and management of AEs, weekly study nurse joint meetings (JF) are held to review patient feedback and monthly project group meetings are used to discuss all reported AEs, as well as qualitative feedback from participants. If necessary, protocol adjustments are made based on these discussions. All AEs will be documented in the study database, in a free-text field labeled “Special Considerations,” to ensure comprehensive tracking, evaluation, and management of any adverse effects associated with the study interventions or its conduct. This approach ensures that potential harms are promptly addressed, while participant safety and well-being are maintained throughout the study.

### Frequency and plans for auditing trial conduct {23}

Audits are planned once a year after the start of recruitment and are organized by the consortium management.

### Plans for communicating important protocol amendments to relevant parties (e.g. trial participants, ethical committees) {25}

Relevant changes to the study protocol are submitted by both the consortium management and all participating centers to the respective ethics committees for approval.

### Dissemination plans {31a}

The study entry in the ClinicalTrials.gov registry is continuously updated. Information about the PRO-P project can be found on the project website https:\\pro-p.info. It is planned to continuously update the website and present study-related events as well as layperson-friendly study results on the website. At the conclusion of the study, a kickoff meeting is planned, to which both healthcare providers as well as patients, and patient representatives will be invited. The preliminary study results will be presented and discussed during this meeting. Study results are presented at scientific conferences and preferably published open-access Table

## Discussion

This is the first multicenter randomized controlled study investigating the effectiveness of postoperative electronic patient-reported outcome measures (ePROMs) following surgical intervention (radical prostatectomy for localized prostate cancer). We anticipate a significant reduction in the physical symptom burden, primarily urinary incontinence, and psychological symptom burden in the intervention group, accompanied by improvements in patient competence and health-related quality of life.

The process evaluation based on the perspectives of both patients and healthcare providers is expected to provide insights into facilitating factors as well as barriers in the implementation, execution, and adoption of ePROMs in this context.

The structured follow-up care within the framework of the PRO-P study closes a gap in care in the follow-up and treatment of physical and psychological consequences of radical prostatectomy.

The implementation of new digital applications remains a challenge, taking into account the interfaces with different hospital information systems at the prostate cancer centers. At all centers, however, there is already corresponding preparatory work through the PCO study, so only the additional digital survey tools need to be implemented. Another obstacle could be the unwillingness of patients to participate in the electronic collection of data, because of a lack of technical means, low digital health literacy, and/or objections to digital data collection. Language barriers among patients could possibly lead to a lack of willingness to participate. This has already been taken into account when calculating the number of cases. However, older patients nowadays widely use smartphones or desktop computers. Furthermore, study nurses at the prostate cancer treatment centers can provide additional assistance.

### Utilization potential

The relevance for the health care system lies in early detection, counseling, and treatment of distressing postoperative symptoms and improved cross-provider networking between centers and office-based urologists. In the future, follow-up care using ePROMs could be integrated into the legally required electronic patient record. In the long term, electronic follow-up care by means of ePROMs can and should be led and managed by outpatient urology practices.

EHealth interventions may clarify problems and thus resources could be saved. Extrapolation to other areas of health care is possible. Various publications address the inclusion of PROMs in the oncological follow-up of patients with different tumor types [24].

## Conclusion

In light of existing efficacy indications for breast cancer, colorectal cancer, and advanced malignancies, this study represents a pioneering investigation into symptom monitoring for prostate cancer, particularly in the context of primary surgical intervention (radical prostatectomy). Anticipated outcomes include a reduced symptom burden and improved quality of life for prostate cancer patients. Expectations encompass gaining valuable insights into the intervention's mechanism of action, its further refinement, and elucidation of inhibitory and facilitative factors influencing the implementation of the intervention.

## Trial status

Study protocol version 2.3, dated May 10, 2023. Recruitment started on April 01, 2023. The end of recruitment is planned for July 31, 2024. The end of data collection/last visit is planned for August 31, 2025. The first results are expected in December 2025*.*

Due to the complexity of the technical infrastructure and the corresponding need for ongoing coordination with the data protection institutions, an extended technical development period and repeated adjustments to the study protocol were necessary, meaning that recruitment could only start later than originally planned. Another consequence was that the study protocol could only be submitted late in the recruitment process.

## Supplementary Information

Supplementary Material 1. PRO-P SPIRIT checklist.

## Data Availability

The final study data set will be located at the IMSB of the University of Cologne and the final quantitative data at the IMVR of the University of Cologne. The handling of and access to the data is regulated in a cooperation agreement including a joint control agreement and a publication agreement between all partners. Reasonable requests for access to the data can be addressed to the corresponding author and will be decided by the Steering Board.

## Abbreviations

DKG: Deutsche Krebsgesellschaft (German Cancer Society)
eCRF: Electronical Case Report Form
EPIC-26: Expanded Prostate Cancer Index Composite
ePROMs: Electronic patient-reported outcome measures
EQ-5D-5L: European Quality of Life Five-Dimension Questionnaire
GCP: Good Clinical Practice
HIS: Hospital Information System
MID: Minimally important difference
PC: Prostate cancer
PCO: Prostate Cancer Outcomes Study
PEI: Patient Enablement Instrument
PHQ-4: Patient Health Questionnaire
PSA: Prostate-specific antigen
QoL: Quality of life
